# Molecular epidemiology and risk factors associated with *Babesia spp* infections in cattle in Imbo region, Burundi

**DOI:** 10.1007/s12639-025-01871-7

**Published:** 2025-10-24

**Authors:** Eric Ndayikengurukiye, Jean Bosco Ntirandekura, Lionel Nyabongo, Amos Amore, Francis McOdimba

**Affiliations:** 1 Animal Production Program, Research Department, Institut des Sciences Agronomiques du Burundi (ISABU), P.O Box 795, Bujumbura, Burundi; 2 Department of Biological Sciences, Faculty of Sciences, Egerton University, Njoro, Nakuru, Kenya; 3 Department of Animal Health and Productions, University of Burundi, P.O Box 2940, Bujumbura, Burundi; 4 International Livestock Research Institute, ILRI Burundi Office, Bujumbura, Burundi; 5 International Livestock Research Institute, ILRI Tanzania Office, Dar es Salaam, Tanzania

**Keywords:** *Babesia bovis*, *Babesia bigemina*, Prevalence, Cattle, Risk factors

## Abstract

Bovine Babesiosis is one of the tick-borne diseases (TBD) caused by *Babesia spp* and is transmitted by tick vectors of the genus *Rhipicephalus microplus*. The disease causes significant economic loses to livestock farmers and it has been reported to be endemic in many countries of East African region including Burundi. A recent study conducted in Burundi showed that the vectors of the disease are present in different agro-ecological zones. A cross-sectional study was conducted in the Imbo region to assess the prevalence and risk factors associated with bovine babesiosis. A total of 400 blood samples drawn from cattle were collected from either jugular or coccinea vein. Nested Polymerase Chain Reaction (nPCR) assay based on rhoptry associated protein-1a (*Rap-1a)*, and spherical body protein-4 (*SBP-4)* was used for pathogen detection and the resulting data was used to determine the risk factors using the R statistical software. Gene sequences and the genetic characterization were determined for selected positive samples. Out of a total of 400 samples, 5.50% (3.3–7.8, 95% CI) were found to be infected with *Babesia spp* where 4.25% (2.3–6.2, 95% CI) and 1.25% (0.2–2.3, 95% CI) were infected with *B. bigemina* and *B. bovis* respectively. Age, sex, tick infestation, grazing system, and origin (commune) were found to be significantly associated with bovine babesiosis (*P* value < 0.05). The sequence analysis revealed that different genotypes of *B. bigemina* and *B. bovis* are present in the Imbo region and these results will help the competent authorities to design an effective strategy for the control of tick-borne diseases to reduce the economic losses causes by the diseases.

## Introduction

Babesiosis (also known as piroplasmosis, Texas fever, or red water) is a tick-borne disease (TBD) caused by *Babesia spp* and can infect many domestic and wild animals and occasionally humans (Krause 2019; OIE 2021)*.* While *Babesia species* such as *B. bovis*, *B. bigemina* and *B.divergens* are well-known pathogens in cattle, (Antonio Alvarez et al. 2019) other species like *B. occultans* have been reported to exhibit varying degrees of pathogenicity depending on the region (Noaman et al. 2021) Livestock acquire infection through the bite of infected ticks where the sporozoites enter through the site of bite and are discharged into circulation (Chandran 2021). In humans, it can also be transmitted through blood transfusion, from the mother to the foetus via the placenta or through organ transplants (Krause 2019; Shen and Liu 2019). Human babesiosis is considered an emerging zoonotic disease and has a worldwide distribution (Puri et al. 2021).

In cattle, the disease clinically manifests with different signs including hyperthermia (> 40 °C), anorexia, jaundice, haemoglobinuria, dyspnoea, pale mucus membrane, reduction of rumination and milk production and abortion in pregnant cows may also occur (Menshawy 2020; Chandran 2021; Almazán et al. 2022; Fesseha et al. 2022). Laboratory diagnosis includes several techniques such as microscopy, serological tests and molecular techniques (Polymerase Chain Reaction (PCR) (Antonio Alvarez et al. 2019; OIE 2021).

Economic losses due to bovine babesiosis include mortality, morbidity, reduced meat and milk production, abortion and expenses related to the treatment of infected animals and the control of the ticks vector (Rodríguez-Vivas et al. 2017; Jabir 2017; Enbiyale et al. 2018). The breed, sex, age, season, presence of ticks and the farming system (intensive or extensive) are the major risk factors that are associated with the prevalence of babesiosis in cattle (Swai et al. 2007; Franck et al. 2017; Chiuya et al. 2021).

Generally bovine babesiosis occurs in all continents of the world wherever ticks (*Rhipicephalus* spp) which are vectors of this disease exist (Marques et al. 2020) and it is mostly common in tropical and subtropical regions. *B. bigemina* and *B. bovis* are common in South and Central America, Europe, Africa, Middle East, Central Asia and Australia. (Jabir 2017; MacGregor et al. 2021). In Africa, babesiosis is caused mainly by two species of the apicomplexan protozoa, including *Babesia bigemina* and *Babesia bovis* (Adjou Moumouni et al. 2015)*.* The prevalence of bovine babesiosis varies from one continent to another, from one country to another or from one region to another. The overall prevalence of bovine babesiosis worldwide is estimated at 29% while sero-prevalence is estimated at 50% (Jacob et al. 2020). In Burundi in general, and specifically in the Imbo region, there is a lack of information on the epidemiology of bovine babesiosis. Therefore, this study was undertaken to assess the prevalence and risk factors associated with babesiosis in cattle and to determine the genetic diversity of *Babesia spp* in Imbo Region in Burundi.

## Materials and method

### Study area

**Fig. 1:**
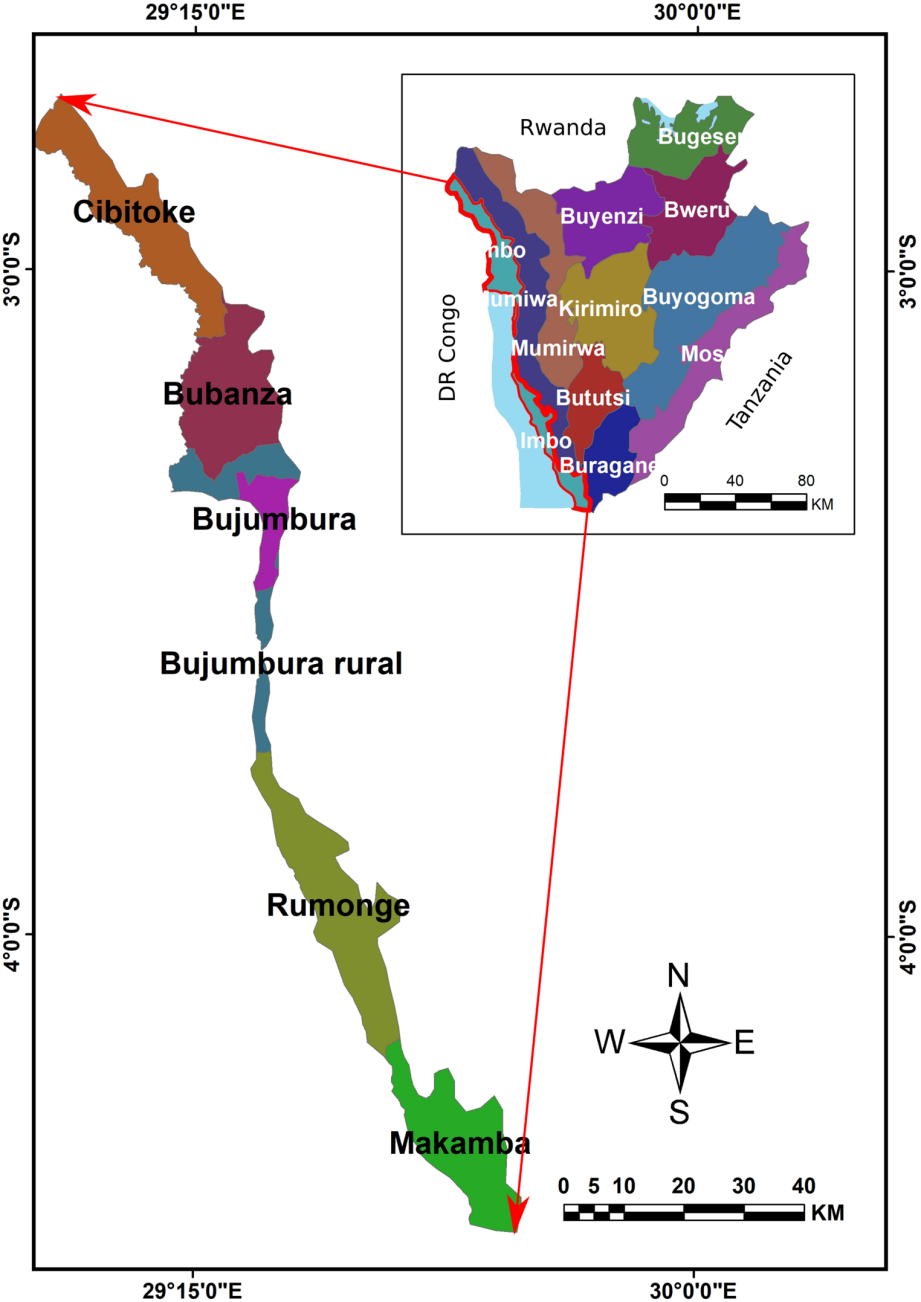
Map showing the Imbo region of Burundi and its six provinces (Nkurunziza et al. 2024)

This study was conducted in 5 different communes located in 5 provinces of Imbo region of Burundi. These communes are namely Rugazi, Mutimbuzi, Rugombo, Nyanza-lac and Rumonge and are in Bubanza, Bujumbura Rural, Cibitoke, Makamba and Rumonge provinces respectively. The Imbo Region extends unequally over six provinces, namely Bubanza, Bujumbura Rural, Bujumbura Mairie, Makamba and Rumonge and comprises 11 rural communes and 3 urban communes of the city of Bujumbura. This region is located between 2°48′30″ and 4°20′43″ latitude South and 29°36′3″ longitude east. It is the westernmost region and extends Northwards from Lake Tanganyika towards the Democratic Republic of Congo (Fig. 1). The region is characterized by annual rainfall of 800 mm to 1100 mm spread over 7 to 8 months. This region is the most densely populated part of Burundi with 300 inhabitants/km^2^ and the livelihood of the population is largely dependent on agriculture and livestock farming (MEETU 2013).

### Study design and sample size

This study was a cross-sectional study conducted in the Imbo region from September to December 2022. The prevalence of babesia parasites was determined using Nested PCR (nPCR) from the blood samples collected in the farms from either the jugular or coccygeal vein. Five millilitres of blood samples were collected in labelled Ethylenediaminetetraacetic Acid (EDTA) tubes and transported in cool boxes with ice to the National Veterinary laboratory at Bujumbura and stored at − 20 °C prior to sending them to the Biosciences eastern and central Africa-International Livestock Research Institute (BecA-ILRI) hub, Nairobi for molecular screening by PCR to determine the presence of *B. bigemina* and *B. bovis* and for sequencing of the positive samples.

During blood samples collection, information on animal characteristics including sex, age, breed, body condition scores (BCS), origin (communes and provinces), tick infestation, tick control and grazing system were recorded using a web-based mobile phone application, Kobocollect (Version May 20, 2018, Copyright Act of 1998, Sect. 512 of the U.S. Copyright Act) to determine the risk factors associated with bovine babesiosis. The age of animals was estimated based on the information provided by the owners and completed as was described by Hutu (2020). The sampled cattle were selected purposively based on the conditions listed below, in the households located in 5 provinces involving 5 communes including 20 hills in the Imbo region. The farmers surveyed were either the cattle owners or household heads. In their absence, other adult members of the family, 18 years old and above, were interviewed. For each household, one or more cattle was sampled depending on the herd size and the inclusion criteria, such as cattle that were more than 10 months old and those having at least one tick on their body or cattle with the following clinical signs: anaemia, muscle tremors, haematuria, jaundice, dyspnoea, pale mucus membrane and weight loss.

A multistage random sampling strategy was used for selecting farmers for the study. At the beginning of the study, the local veterinary services were contacted to obtain the list of the provinces, communes, and hills of the Imbo region. Local veterinary services of the hills, communes and provinces selected were consulted to obtain the list of cattle owners on each hill. Based on this list, the names of provinces, communes, hills, cattle owners visited were randomly generated using the RAND function in Microsoft Excel. Since there was no previous study in Burundi to establish the prevalence of bovine babesiosis, the sample size was determined by taking the prevalence of 50%, absolute, precision of 5%, and confidence interval level of 95% using the formula as reported by (Abdela et al. 2018).

Sample size of cattle:

$$\mathrm{N}=\frac{{1.96}^{2}Pexp\left(1-Pexp\right)}{{d}^{2}}=\frac{{1.96}^{2}*0.5*\left(1-0.5\right)}{{0.05}^{2}}=384$$

where N = required sample size; 1.96 t = the value of Z at 95% confidence interval; P exp = Expected prevalence = 50% (0.5); d = desire absolute precision = 5% (Standard value of 0.05).

According to the formula, a total of 384 animals were required to be sampled, but 400 animals were used to increase the degree of precision in 5 provinces. 

### DNA extraction

Genomic DNA was extracted from each sample using 300 μl of the whole blood using the TANBead^R^ Nucleic Acid Extraction commercial kit (W6T2A46) according to the manufacturer’s instructions, on the TANBead Nucleic Acid Extractor Maelstrom 9600 machine for Deoxyribonucleic acid (DNA) extraction. The quantity and purity of extracted DNA samples were measured with Thermo NanoDrop 800 spectrophotometer, (Thermo Fisher Scientific, Waltham, MA, USA). After extraction, the DNA was stored at− 20^o^ C pending PCR analysis.

### PCR and nested PCR

Specific primers targeting *B. bovis* spherical body protein-4 *(SBP-4),* and *B. bigemina* rhoptry associated protein-1a (*RAP-1a*), were used to amplify the respective genes (Adjou Moumouni et al. 2015). The PCR amplification was done in a 10 μl-reaction mixture having 2 μl of DNA template, 0.5 μl of each forward and reverse primers, 5 μl of One Taq 2X Master Mix (with standard buffer and 2 μl of nuclease free-water). The thermocycling conditions for PCR amplification were as follows: initial denaturation-2 min at 94 °C followed by 40 cycles (30 s of denaturation at 94 °C, 30 s of annealing at 55 °C, and 1 min of extension at 72 °C) and final extension at 72 °C for 10 min using Thermal cycler (Master cycler Nexus GSX1). The nested PCR was done using 1 μl of each sample from the first PCR product. The 1 μl of the first PCR product of each sample was diluted in 9 μl of water before the second amplification. The second PCR amplification was done in a 10 μl reaction mixture having 1 μl of DNA template, 0.5 μl of each primer (forward and reverse primers), 5 μl of One Taq 2X Master Mix (with standard buffer and 3 μl of nuclease free water) The thermocycling conditions were as follows: initial denaturation in 2 min at 94 °C followed by 40 cycles (30 s of denaturation at 94 °C, 15 s of annealing at 55 °C, and 45 s of extension at 72 °C) and final extension at 72 °C for 10 min. For each PCR run, DNAse free water was used as negative control while DNA samples of *B. bovis* and *B. bigemina* which had been confirmed as positive were used as positive controls. For each of the assays, the nested PCR products were electrophoresed on a 1.5% agarose gel, stained with ethidium bromide and visualised under UV light (Azure biosystems C280) Samples showing a band of similar size (412 base pairs for *B. bigemina* and 503 base pairs for *B. bovis*) with the positive control were considered positive for the corresponding pathogen.

### Sequencing of positive samples

All positive samples (*B. bigemina*, n = 17 and *B. bovis*, n = 5) were selected for DNA sequencing. Purification of the DNA samples was done using Exo-CIP Rapid PCR Cleanup Kit E1050L according to the manufacturer’s instructions. The cleaned PCR products were treated for sequencing using the reaction sequencing components including BigDye, pGEM, 5X sequencing buffer, distilled water, Template DNA, and the specific primers targeting spherical body protein-4 *(SBP-4)* for *B. bovis* and rhoptry associated protein-1a *(RAP-1a)* for *B. bigemina* according to the manufacturer’s instructions and protocol. The total reaction volume of 10 µl was run with PCR cycling protocol as follows: an initial denaturation at 96 °C for 2 min; followed by 30 cycles of denaturation at 96 °C for 10 s, annealing at 50 °C for 10 s, extension at 60 °C for 4 min, and followed by final extension at 72 °C for 4 min and a hold at 4 °C ∞ using Thermal Cycler (GeneAmp PCR System 9700). The PCR products were treated for sequencing using 3730 Buffer (10X) with EDTA, absolute ethanol 98.8% and Hide^HT^ formamide according to the manufacture’s instruction and protocol. Each of the purified samples was sequenced using the 3730 DNA analyser, (Sanger sequencing machine, Applied Biosystems) using both the forward and reverse primers.

### Sequence and phylogenetic analysis.

The bioinformatics analysis for the samples sequenced was done by generating the consensus sequence for each sample, using Geneious Prime 2023.2 tool (https://www.geneious.com). To create a single consensus sequence, the Geneious de novo assembler was used to align pairs of forward and reverse Sanger sequences with the error probability limit of 0.05 after trimming the poor-quality sequence. All the fasta consensus files were concatenated to perform the alignment by using MAFFT version 7 (Katoh and Standley 2013), a multiple sequence alignment tool. To remove spurious sequences or poorly aligned regions from the sequences alignment, TrimAl (Capella-Gutiérrez et al. 2009) was used with the default parameters. The *B. bovis* and *B. bigemina* sequence obtained in this study were submitted using the Banklt tool (https://www.ncbi.nlm.nih.gov/WebSub/?form=history&tool=genbank: accessed on 23rd October 2024), to get the access number compared for nucleotides identity with the others sequence from other countries available in NCBI GenBank database. The access number of our sequence are PQ483198, PQ483199, PQ483200, PQ483201 for *B. bigemina* and PQ496639, PQ496640, PQ486641for *B. bovis.*

### Data analysis

The data obtained during the laboratory analyses (PCR) as well as the survey data and information on the animals from which the samples were obtained, were entered into the Microsoft Excel database (Microsoft Corp, Redmond, WA, USA). The data were imported into the statistical software R (v4.2.2) for analysis and the prevalence, and standard deviation were generated. Chi-square and odds ratio were also calculated to assess the association between bovine babesiosis and breed, sex, age, body condition, presence of ticks, tick control, grazing system, and origin (Commune). A 95% confidence interval and a *P* value less than 0.05 (at the 5% significance level) were considered significant in all analyses.

For sequencing analysis, the *B. bovis* and *B. bigemina* sequence obtained in this study were submitted using the Banklt tool (https://www.ncbi.nlm.nih.gov/WebSub/?form=history&tool=genbank:), to get the access number and were used to compare nucleotides identity with the others sequence from the same pathogen and other countries available in NCBI GenBank database (https://www.ncbi.nlm.nih.gov/: accessed on 26th November 2024), and all were used in phylogenetic tree construction using MEGA v.7.0. The maximum-likelihood method was used for genetic analysis of *B. bigemina (RAP-1a)* and *B. bovis (SBP-4) gene* and the bootstrap consensus tree inferred of 1000 was taken to represent the evolution history of the taxa analysed (Tamura et al. 2004).

## Results

### Prevalence of bovine babesiosis

A total of 400 cattle in Imbo region were included in this study, 103 from Rugombo, 63 from Mutimbuzi, 96 from Rugazi, 81 from Nyanza Lac and 57 animals from Rumonge. In total, the laboratory result showed that 22 (5.50%, CI 3.3–7.8) cattle were infected with *Babesia spp* where 17 (4.25%, CI 2.3–6.2) and 5 (1.25%, CI 0.2–2.3) cattle were positive for *B. bigemina* and *B. bovis* respectively (Table 1). There were no coinfections with both species. Following the laboratory analyses, based on the origin (commune) of the cattle blood samples, the results showed that one sample (1.59%) in Mutimbuzi commune of Bujumbura Rural province, two samples (1.94%) in Rugombo commune of Cibitoke province, three samples (3.12%) in Rugazi commune of Bubanza province, four samples (7.2%) in Rumonge commune of Rumonge province and 12 samples (14.81%) in Nyanza lac of Makamba province had bovine babesia parasites (Table 2). The origin was found statistically associated with bovine babesiosis with *P *value = 0.0002, *χ2* = 21.3 (Table 2).

**Table 1:** Prevalence of *Babesia sp*

Test	*Babesia specie*	Sample size	Positive	CI
PCR	*Babesia bigemina*	400	17 (4.25%)	(2.27–6.23)
	*Babesia bovis*	400	5 (1.25%)	(0.16–2.34)
	*Total*	400	22 (5.50%)	(3.3–7.8)

PCR, polymerase chain reaction; CI, confidence interval

**Table 2:** Prevalence of bovine babesiosis based on the origin of the animals in the Imbo region

Origin of sample	Number of cattle examined	-ve	+ ve	Prevalence % (95% CI)	χ2	*df*	*P* value
Mutimbuzi	63	62	1	1.59 (− 1.50, 4.67)	21.639	4	0.00023
Nyanza lac	81	69	12	14.81 (7.08–22.55)			
Rugazi	96	93	3	3.12 (− 0.36–6.61)			
Rugombo	103	101	2	1.94 (− 0.72–4.61)			
Rumonge	57	53	4	7.02 (0.39–13.65)			

-ve, negative; + ve, positive; 95% CI, confidence interval, χ2, chi-square; *df*, degree of freedom

### The risk factors associated with bovine babesiosis.

The different risk factors including age, sex, breed, body corporal score (BCS), tick infestation, tick control, grazing system, and origin (commune) were considered in univariable and multivariable logistic regression analysis. Among these risk factors age, sex, tick infestation, grazing system, and origin (commune) were found to be significantly associated with bovine babesiosis (*P *value < 0.05) in univariable logistic regression analysis (Table 3). On the other hand, BCS and tick control were not associated with bovine babesiosis (*P* value > 0.05). Using the multivariable logistic regression analysis, none of these factors considered was found to be significantly associated with bovine babesiosis (*P* value > 0.05) (Table 4).

**Table 3:** Univariate analysis of risk factors associated with bovine babesiosis

Risks factors	Cattle tested	− ve	+ ve	Prevalence % (95% CI)	OR	χ2	*df*	*P *value
Age						8.8709	2	0.01185
Young	95	84	11	11.58 (5.14–18.01)	3.61 (1.35–9.64)			
Adult	200	193	7	3.50 (0.95–6.05)	ref			
Old	105	101	4	3.81 (0.15–7.47)	0.69 (0.19–2.43)			
Sex						6.4085	1	0.01136
Male	55	48	7	12.73 (3.92–21.54)	3.21 (1.24–8.27)			
Female	345	330	15	4.35 (2.20–6.50)	Ref			
Breed						6.99	2	0.03
Local	78	69	9	11.54 (4.45, 18.63)	3.44 (1.28–9.26)			
Crossbred	103	98	5	4.85 (0.70, 9.00)	1.35 (0.43–4.22)			
Exotic	219	211	8	3.65 (1.17, 6.14)	Ref			
Body condition						1.87	2	0.39
Poor	27	24	3	11.11 (-0.74, 22.97)	2.62(0.61–11.22)			
Medium	132	126	6	4.55 (0.99–8.10)	Ref			
Good	228	228	13	5.39 (2.54–8.25)	1.20 (0.44–3.23)			
Tick infestation						9.8642	1	0.0016
Yes	45	38	7	15.56 (4.97–26.14)	4.18(1.60–10.88)			
No	355	340	15	4.23 (2.13–6.32)	Ref			
Tick control						2.7981	1	0.09
Regular	297	284	13	4.38 (2.05–6.70)	Ref			
Irregular	103	94	9	8.74 (3.28, 14.19)	2.09 (0.87–5.05)			
Grazing system						13.414	2	0.0012
Free	30	24	6	20.00 (5.69–34.31)	5.41(1.94–15.07)			
Semi zero	8	8	0	0	0			
Zero	362	346	16	4.42 (2.30–6.54)	Ref			

-ve, negative; + ve, positive; 95% CI, Confidence interval; OR, odds ration; χ2, chi-square; *df*, degree of freedom

**Table 4:** Multivariate logistic regression model analysis of risk factors

Variable	β-coefficient	*P *value
Intercept	0.599470	0.8105
Age of the animal	− 0.438916	0.2373
Sex of the animal	− 0.810456	0.1661
Breed of the animal	− 0.151033	0.6536
Body condition	0.001641	0.9965
Tick infestation	− 0.264488	0.7693
Tick control	− 0.90467	0.8894
Grazing system	− 0.500879	0.2657
Origin (commune)	0.341218	0.0808

Our study showed that the prevalence of bovine babesiosis based on the age and sex was higher in young animal < 3 years (11.58%) than old > 6 years (3.81%) and adult 2–6 years (3.5%); and was higher in male (12.73%) than in female (4.35%). The univariate logistic regression analysis showed that the risk factors like age (*P* value = 0.01, *χ2* = 21.9) and sex (*P *value = 0.01, *χ2* = 6.4) (Table 3) were statistically associated with the prevalence of bovine babesiosis. However, the young animals < 3 years (OR 3.61, CI 1.35–9.64) and male (OR 3.21, CI 1.24–8.27) were significantly more likely to be at high risk of contracting bovine babesiosis (Table 3).

The study showed that the prevalence of bovine babesiosis based on the breed of animal and tick infestations was high in local breed (11.54%) than crossbreed (4.85%) and pure breed (3.65) and was higher in animals with tick infestation (15.56%) compared to animals without tick infestations (4.23%). The univariate analysis using *χ2* test showed the risk factor like breed (*P *Value = 0.03, χ2 = 6.7) and tick infestation (*P *value = 0.02, χ2 = 9.9) were significantly associated with the prevalence of bovine babesiosis infection and the local breed (OR = 3.44, CI 1.28–9.26) and the animal with tick infestation (OR = 4.18, CI 1.60–10.88) were more likely to be at risk of get bovine babesiosis in this region (Table 3).

The univariate logistic regression analysis showed that the risk of babesia infection was insignificantly higher in animals with poor BCS (11.11%) than medium BCS (4.55%) and good BCS (5.39%) (*P *value = 0.4, χ2 = 1.9). The prevalence of bovine babesiosis based on the grazing system was higher in the free system (20.00%) than in zero (4.2%) and in semi-zero (0%) grazing system. The univariate analysis using χ2 test showed the grazing system (*P* Value = 0.001, χ2 = 13.41) was significantly associated with the prevalence of bovine babesiosis and the animal in free grazing system (OR = 5.41, CI = 1.94–15.07) was more likely to get bovine babesiosis (Table 3).

### Comparative gene sequence analysis

The *B. bovis* and *B. bigemina* sequence obtained in this study were submitted using the Banklt tool (https://www.ncbi.nlm.nih.gov/WebSub/?form=history&tool=genbank: accessed on 23rd October 2024), to get the access number compared for nucleotides identity with the others sequence from other countries available in NCBI GenBank database. The access number our sequence are PQ483198, PQ483199, PQ483200, PQ483201 for *B.bigemina* and PQ496639, PQ496640, PQ486641for *B.bovis* and were compared for nucleotides identity with sequences from other countries available in NCBI GenBank database. Compared with sequence available in blast (https://blast.ncbi.nlm.nih.gov/Blast.cgi?PROGRAM=blastn&PAGE_TYPE=BlastSearch&LINK_LOC=blasthome: accessed on 25th November 2024), the *B.bigemina (RAP-1a)* gene (sequence1, sequence 4) shared 100% nucleotide similarities with sequences from Tanzania (OP390283, MG 210823, MG210824, MN807306, MN 807309), Kenya (KP347559), Uganda (MG426200, MG426202) and (MH265105), South Africa (MK481015), Malawi (OP86671). Furthermore, the *B. bovis (SBP-4)* sequences (sequence 1, 2, and 3) showed 99.42–100% nucleotide similarities to sequences from South Africa (KF626631, KF626632, KF626634), Benin (KX685402), Egypt (MZ197893, KF192805), Brazil (AB569300), Thailand (AB594814).

**Fig. 2:**
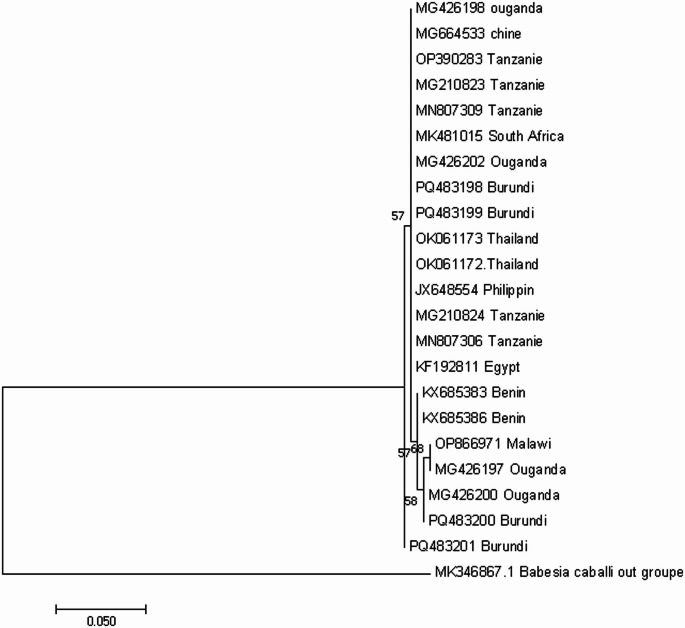
Phylogenetic analyses of B. bigemina *RAP-1a* gene sequences obtained from Imbo region Burundi. B. cabali was used as out group. The tree was constructed by MEGA ver.7 using the maximum likelihood method using the kimura 2 parameters model confidence of occurrence of the nodes was assessed by bootstrap in 1000 replications

**Fig. 3:**
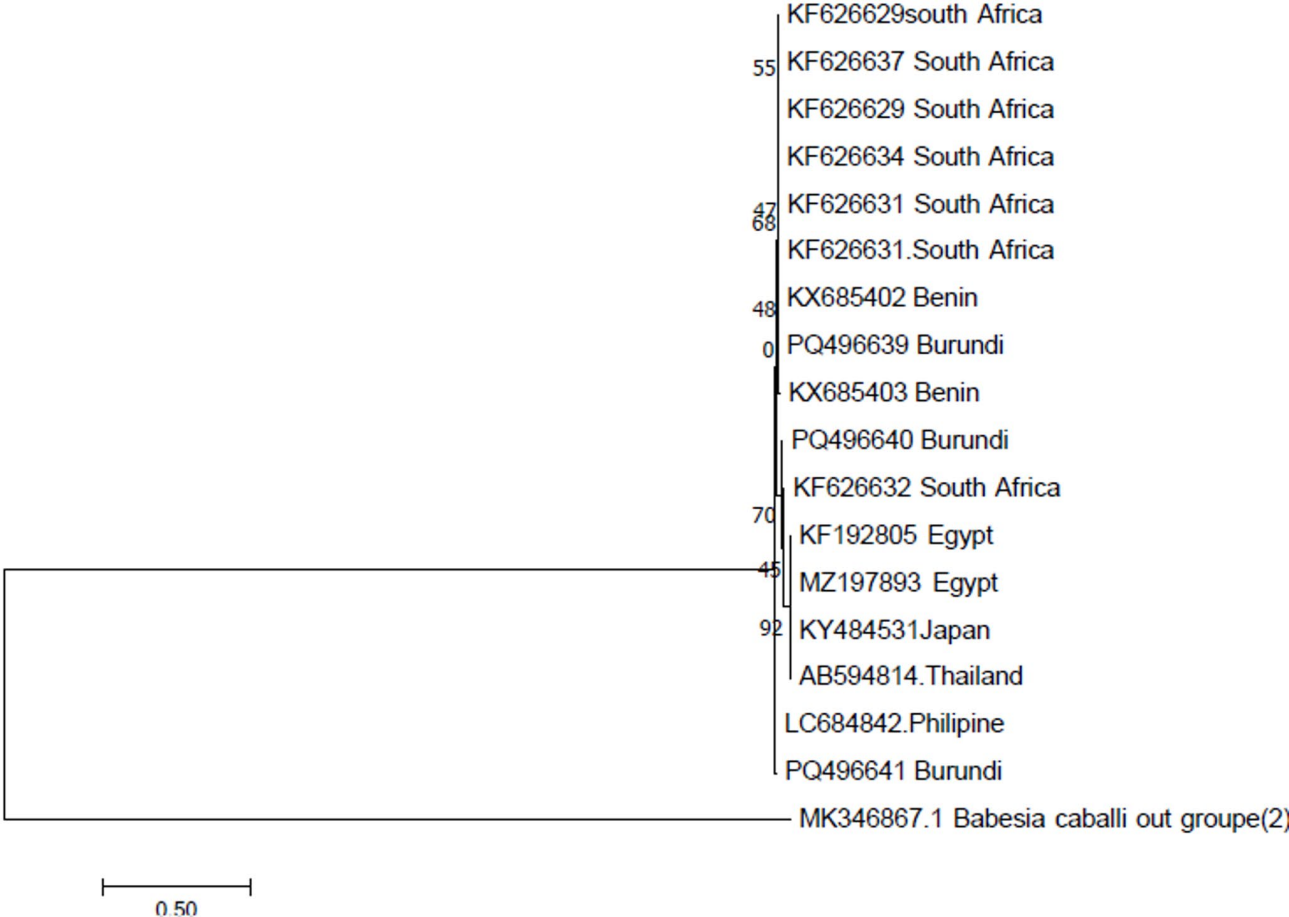
Phylogenetic analyses of B. bovis *SBP-4* gene sequences obtained from Imbo region Burundi. B. Cabali was used as out group. The tree was constructed by MEGA ver.7 using the maximum likelihood method using the kimura 2 parameters model confidence of occurrence of the nodes was assessed by bootstrap in 1000 replications

The phylogenetic tree of *B. bigemina* and *B. bovis* was constructed based on the gene *RAP-1a* and *SBP-4* respectively, together with other genes extracted from NCBI GenBank database (https://www.ncbi.nlm.nih.gov/: accessed on 26th November 2024),). The sequence of the gene *RAP-1a* were clustered in two clusters (Fig. 3), some sequences appeared in the same clade together with the sequence MG21O823, MN807309 from Tanzania and MG426202 from Uganda and other appeared in the same clade with the sequence MG426200, MG426197 from Uganda and OP866971 from Malawi (Fig. 2). Moreover, the sequence of *B. bovis*, in this study was also clustered in two clusters. The first group appeared in the same clade together with the sequences, LC611417 from South Soudan, KF626631from South Africa and KF192805 from Egypt while the second group appeared together with the sequences KX685402, KX685403 from Benin and KF626629, KF626634, KF626637 from South Africa (Fig. 3).

## Discussion

*Babesia bovis* and *Babesia bigemina* are the two species that cause numerous economic losses among the livestock keepers in many sub-Sahara countries (MacGregor et al. 2021). The results of the current study showed that the overall prevalence of bovine babesiosis using PCR was 5.50%, (Table 1). These results are in agreement with the study of Namomsa et al. (2023) in East Wollega zone in Ethiopia that found that the prevalence was 5.2%. However, the current prevalence in the area of study was lower than the prevalence reported in Zanzibar Island in Tanzania with 7.30% (Ringo et al. 2020) and to many studies conducted in different parts of Africa. For example, in Kwale county in Kenya the prevalence was found to be 25.8% (Githaka et al. 2021, 2022) Around Jimma Town in South western Ethiopian the prevalence was found to be 11.7% (Abdela et al. 2018) In Gairo and Moduli in Tanzania the prevalence was found to be 14% (Haji et al. 2022) while in Central and Eastern Uganda it was found to be 17.2% (Tayebwa et al. 2018). The differences of prevalence in those previous studies and the current study could be attributed to a number of factors including i) the farming practices where the sampled animals came from. Most of the animals sampled in those studies were in free range extensive grazing system where they had more chance to be in contact with ticks (Kasaija et al. 2021) while most of the animals sampled in this study were from intensive system (zero grazing system) ii) in this study the blood sampling collection was done after a long dry season. Normally, in Burundi the dry season is 3 months (June, July and August) (MEEATU 2013) but in 2022 the rainy season came later in October and when the temperature stayed higher for a long period (dry season). It had been reported that when the temperatures stay high for a long period, it can inhibit and eventually eliminate the *B. bigemina* from the tick (Kocan 1995; Ringo et al. 2020) iii) the higher temperature can reduce the population of ticks since the ticks multiply in places with optimum temperature and humidity (Eisen 2008; Estrada-Peña and De La Fuente 2014).

On the other hand, the prevalence in this study was higher than the prevalence reported in western Kenya (Busia, Bungoma and Kakamega counties) at 0.2% (Chiuya et al. 2021). The prevalence of bovine babesiosis found in the current study was also higher than the 1.5% prevalence found by Disassa et al. (2015) in western Ethiopian. The lower prevalences in many studies could also be attributed to the sensitivity of the tests used in diagnosis, since many of these studies used microscopy and serological techniques which are less sensitive and cannot detect low parasitaemia (OIE 2021). In a recent study conducted in Burundi, Nyabongo et al. (2021) reported a seroprevalence between 30 and 90% of *B. bigemina* while there was no case of *B. bovis* reported. The difference of the two prevalence values can be explained by the sensitivity and the specificity of the test used in the current study. In additional, as the ELISA test check for antibody against the parasite, cannot make the difference between the current and old infection that’s why many time you find that the seroprevalence is higher (Antonio Alvarez et al. 2019; Ganzinelli et al. 2020; OIE 2021).

In the current study the prevalence of *B. bigemina* was higher than that of *B.bovis* (Table 1) which is in agreement with the studies reported by Ringo et al. (2020) in Tanzania where the prevalence of *B. bigemina* and *B. bovis* were 5.2% and 2.1% respectively, (Adjou Moumouni et al. 2018) in Benin where they found 20.3% for *B. bigemina* and 11.6% for *B. bovis.* Many other studies agree with our finding where they found that *B. bigemina* is more prevalent than *B. bovis*; Simking et al. (2014) in Salakpra–Thailand; Sibusiso Mtshali and Mtshali (2014) in South Africa, Matheus et al. (2019) in Ohangwena region in Namibia. According to Ganzinelli et al. (2020) the low-level detection of *B. bovis* could be due to the fact that the erythrocytes with *B.bovis* are few in peripheral capillaries, resulting in a lower level of detection of *B. bovis* than that of B. bigemina.

We report here that bovine babesiosis is prevalent in all communes of the study areas in Burundi, but the higher prevalence was found in Nyanza lac commune of Makamba province. This higher prevalence in Nyanza-Lac could be attributed to the grazing system practised by the famers in this area, since most of the farmers visited practise extensive system and the cattle in this system have many chances to be in contact with the vectors (ticks) of babesiosis. In the present study, the prevalence of bovine babesiosis has been significantly higher in young animals (< 3 years) followed by the adult and old animal. These results are in agreement with the finding of Zulfiqar et al. (2012) in southern Punjab and Amorim et al. (2014) in Brazil who reported the higher prevalence in young animals than old animal. However our results disagree with Abdela et al. (2018) who reported that babesiosis was more prevalent in old animals followed by adult and young ones. The low prevalence in adult and old animals compared to the young ones is probably due to old animals having been exposed to the pathogens and the vectors for longer which leads them to develop a better protective immunity than young animal (Homer et al. 2000).

 The sex of the animals was also significantly associated with bovine babesiosis, and the higher prevalence had been recorded in males than in females (Table 3). Our results are in agreement with the finding of Fereig et al. (2017) but differs with Azhar et al. (2023) who reported that the females were more likely to be infected by *Babesia spp* than males. The higher prevalence in male animals suggests that they are more susceptible to babesia infection due to the male hormone-like estra-diol-17β and testosterone, which have been shown to negatively affect the host’s effector immune cells, so that infections cause great anaemia and parasitaemia as was reported by Sasaki et al. (2013). In addition, Sasaki also reported that the immune response is lower in males compared to females because the phagocytic activity of macrophage and productions of inflammatory cytokines is higher in females, and the production of antibody is therefore higher in females than in males.

 Our study also found that the prevalence of bovine babesiosis is significantly associated with the tick infestation (Table 3). A number of studies including Zulfiqar et al. (2012); Amorim et al. (2014); Jirapattharasate et al. (2016); Masih et al. (2021) and Fesseha et al. (2022), also reported that the prevalence of bovine babesiosis is higher in animals with tick infestation than animals without ticks, thus confirming the role of ticks as vectors of babesiosis. Costa et al. (2013) reported that the animals with tick infestation are 18 times more likely to have Tick Borne Diseases (TBD), such as babesiosis, East Coast Fever and anaplasmosis than the animals without tick infestation. This study also found that the local indigenous breeds were more likely to have bovine babesiosis than the crossbreeds and exotic breeds (Table 3). Bock et al. (1997) reported that *Bos taurus* (exotic breed and their cross) were less resistant to babesia than *Bos indicus* (local breeds). The higher prevalence in local breeds could be due to the immunological system of Ankole breed which had lower antibodies against *B. bigemina* (Magona et al. 2011). In this study, we found that the prevalence of bovine babesiosis was significantly associated with the grazing system and the higher prevalence was found in the farmers practising the free grazing system (extensive system) followed by the zero grazing (Table 3). This could be explained by the fact that the animals staying in pasture were more likely to be infested by ticks than the animals which are in zero grazing and staying in the stable (Kasaija et al. 2021).

 Our phylogenetic tree analyses showed that sequence of the *RAP-1a* gene of *B. bigemina* were in different clades, (Fig. 2) which suggest that different genotypes of *B. bigemina* strains are present in the cattle population of the study area. Similar results were reported in different countries of eastern Africa such as Tanzania (Ringo et al. 2020), Uganda (Tayebwa et al. 2018) and Kenya (Adjou Moumouni et al. 2015). Based on *RAP-1A* genetic sequences, the phylogenetic tree of *B. bigemina* isolates in this study showed that the Burundian isolates and those from Kenya, Uganda and Tanzania have the same ancestor, which suggests a possible movement of carrier animals infected with the parasites or infested with the *Boophillus* tick vector during the international animal trade. In addition, most exotic dairy breed of cattle in Burundi are often imported from neighbouring countries such as Tanzania, Kenya, Uganda (Nyabongo et al. 2021) which further justifies the fact that the pathogens found in Burundi are similar to those within the region. The sequence of *SBP-4* gene of *B. bovis* was also in different clades, (Fig. 3) suggesting that different genotype strains of *B. bovis* exist in the study area. Similar findings were reported by Adjou Moumouni et al. (2015) in Kenya. In addition, the phylogenetic analysis shows that the strains of *B. bovis* circulating in the study region have the same ancestor with the strain from different geographical zone such as those from Benin, South Africa, Egypt, Brazil and Thailand. The observations of the genetic diversity of *B.bovis* has been reported by (Gano et al. 2024) in Nigeria, (Adjou Moumouni et al. 2015) in Kenya.

## Conclusion

This study conducted in Imbo region revealed that babesiosis is endemic in the study areas of Burundi and different genotypes of *B. bigemina* and *B. bovis* strains are present in the country although livestock owners do not often report cases of babesiosis. The current study confirms the presence of *B. bovis* in Burundi and the data on the prevalence of the diseases is important and will help decision makers to design an effective strategy for the control of tick-borne diseases to reduce the economic losses caused to the livestock owners and to the country in general. Since this study was conducted in one region, it would be prudent to conduct a larger country-wide survey covering other regions to be able to determine the extent of the existence of the disease in Burundi.

## Data Availability

The datasets generated and/or analyzed during the current study are available in my laptop (Eric Ndayikengurukiye).
